# Microplasma Induced Cell Morphological Changes and Apoptosis of *Ex Vivo* Cultured Human Anterior Lens Epithelial Cells – Relevance to Capsular Opacification

**DOI:** 10.1371/journal.pone.0165883

**Published:** 2016-11-10

**Authors:** Nina Recek, Sofija Andjelić, Nataša Hojnik, Gregor Filipič, Saša Lazović, Alenka Vesel, Gregor Primc, Miran Mozetič, Marko Hawlina, Goran Petrovski, Uroš Cvelbar

**Affiliations:** 1 Department of Surface Engineering and Optoelectronics (F4), Jožef Stefan Institute, Ljubljana, Slovenia; 2 Eye Hospital, University Medical Centre, Ljubljana, Slovenia; 3 Institute of Physics Belgrade, University of Belgrade, Belgrade, Serbia; 4 Stem Cells and Eye Research Laboratory, Department of Ophthalmology, Faculty of Medicine, University of Szeged, Szeged, Hungary; 5 Centre of Eye Research, Department of Ophthalmology, Oslo University Hospital, University of Oslo, Oslo, Norway; Universite Toulouse III Paul Sabatier, FRANCE

## Abstract

Inducing selective or targeted cell apoptosis without affecting large number of neighbouring cells remains a challenge. A plausible method for treatment of posterior capsular opacification (PCO) due to remaining lens epithelial cells (LECs) by reactive chemistry induced by localized single electrode microplasma discharge at top of a needle-like glass electrode with spot size ~3 μm is hereby presented. The focused and highly-localized atmospheric pressure microplasma jet with electrode discharge could induce a dose-dependent apoptosis in selected and targeted individual LECs, which could be confirmed by real-time monitoring of the morphological and structural changes at cellular level. Direct cell treatment with microplasma inside the medium appeared more effective in inducing apoptosis (caspase 8 positivity and DNA fragmentation) at a highly targeted cell level compared to treatment on top of the medium (indirect treatment). Our results show that single cell specific micropipette plasma can be used to selectively induce demise in LECs which remain in the capsular bag after cataract surgery and thus prevent their migration (CXCR4 positivity) to the posterior lens capsule and PCO formation.

## Introduction

The applications of cold atmospheric pressure plasmas (CAP) in biomedicine has been growing enormously in the recent years.[1, 2] The CAPs have been applied for stem cell manipulation, cancer, skin treatments, wound healing and the like [3–5] To the best of our knowledge, this is the first to report highly selective use of CAP upon lens epithelial cells (LECs). These cells are responsible for posterior capsular opacification (PCO), which is a major cause of post-operative or secondary visual loss that develops after cataract surgery in approximately 20% of cases within 5 years.[6] Cataract is still the leading cause of blindness worldwide, while PCO is caused by proliferation and migration of LECs remaining in the capsular bag after cataract surgery. The remaining cells can re-colonize the posterior lens capsule which was otherwise cell-free, and therefore, obstruct the visual axis contributing to light scattering and secondary visual loss.

By using *ex vivo* cultured explants from the human anterior portion of the lens capsule (aLC) and visualization by light microscopy, scanning electron microscopy (SEM) and immunofluorescence staining for proliferation and pluripotency markers, we have already shown that human aLC contains LECs that can migrate and proliferate, suggesting a role of aLC-LECs in PCO formation.[7, 8] Such *ex vivo* cultured aLC-LECs may serve as a model for testing different physical and pharmacological agents against PCO development. Herein, the effect of cold atmospheric pressure microplasma jet (μAPPJ) on the LECs morphology and survival is being investigated. LECs have been previously investigated for their mechanical stress-induced contractions.[9] Similar experimental setup was used for the plasma studies as well.

More generally, atmospheric-pressure plasmas (APPs) have become increasingly attractive for different therapies, since plasmas can trigger a complex sequence of biological responses in tissues and cells.[10] Plasma typically contains short-lived free radicals, including reactive oxygen species (ROS) that can induce cell apoptosis, preferably in tumor cells.[11–16] APP is known to abundantly generate radicals [17] and affect the proliferation and migration of human periodontal ligament mesenchymal stem cells. [18] Plasma can also be used without risk of contamination or secondary infection due to their bactericidal properties.[2, 19–26] To move ahead in the further development of actual commercial tools that can be used in hospitals, and in finding novel and perhaps unexpected uses of plasmas, an understanding of the mechanisms of interaction of non-equilibrium gas discharges with living organisms, tissues and cells has become essential.

Dobrynin *et al*. [27] showed that not only ROS generated in plasma are responsible for achieving a desired effect, but also the charged particles (electrons and ions). The mechanism of plasma interaction with cells is complex, owing partially to the complexity of plasma and mainly to the overwhelming complexity of biological processes. Further complexity is added by the presence of medium as well. We hereby describe direct and indirect interaction of microplasma discharge with targeted cells for limitation of secondary effects of plasma to the surrounding cells and environment. The direct plasma treatment is considered, when a single or targeted LEC is treated by microplasma inside a liquid medium, while indirect is when microplasma is positioned outside and on top of the liquid.[10] We hereby treat aLC-LECs directly and indirectly to study the effects plasma particles generated by bulk plasma, created vapours on interfaces and solvated species created by plasma-liquid interactions. For these purpose, the morphological responses of single or targeted cells were monitored immediately after the treatment and after specified incubation times in order to determine the plasma effects on undergoing apoptosis.

## Materials and Methods

### Microplasma treatment

**Fig 1:**
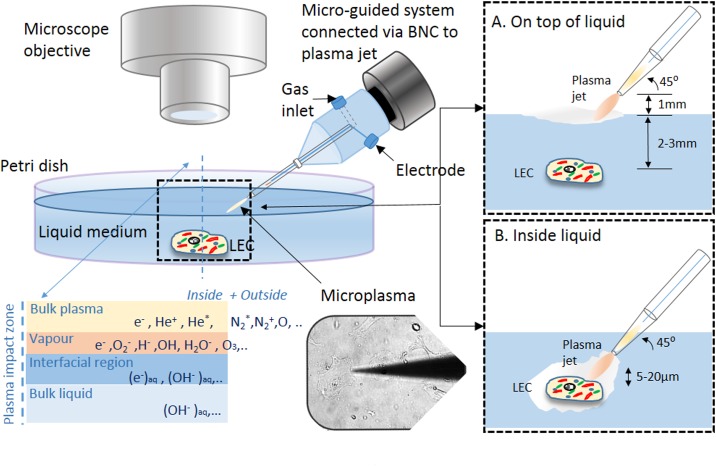
Schematic of the microplasma jet setup with plasma zones and a sketch of the biomedical treatments. A) On the top of the liquid and B) inside the liquid medium.

In order to scale down the plasma volume and achieve a single- or targeted- cell-precision of treatment, modification to the standard APPJ had to be introduced with the electrode configuration.[5, 28, 29] Precision targeted-cell treatment was performed using localized single-electrode microplasma discharge around a needle-like electrode inside the borosilicate glass tube micropipette (no. TW150F-3, World Precision Instruments, USA) with outside diameter 1.2 mm and inner 0.8 mm which had a tip with end opening diameter of ~3 μm. The glass micropipette tip was made by Flaming/Brown micropipette puller (Model P97 Shutter Instruments Co., USA). The experimental setup for targeted-cell-precision microplasma treatment is schematically shown together with some experimental details in Fig 1. The plasma plume was generated around the powered Teflon insulated microelectrode (outer diameter 0.5 mm) with the striped copper wire end of diameter 0.25 mm positioned 2 mm from micropipette end with opening, but still inside the narrowing tip. The generated plasma plum spread visually into air only for about 10–20 μm and thus was suitable for precise treatment of selected cells or larger groups of cells. When the micropipette tip with inner electrode approached the targeted cell(s) in the liquid, the cell membranes served as a floating counter-electrode, while the plasma was brought in a direct contact with its surface; the upper surface of the liquid represented the interface itself. The plasma source [29] used was then powered at 25 kHz, delivering not more than 1 W of real power in all cases. Pure Helium (Messer, He 5.0) was used as the feeding gas and it was flowing between the glass and the insulated electrode copper wire with flow rates adjusted to 0.2 standard litres per minute (slm) by the Matheson FM-1000 mass flow controller. An adapted electrophysiological micromanipulator (model MP 285, Sutter, USA) was used to control and manipulate the microtip with blowing plasma position with the precision of a few micrometers, which made it possible to potentially agitate selected cell area. Adherent cells were selected after careful observation under the microscope.

Two different ways of targeted cell treatment were performed: (i) the selected cell(s) were treated with the microplasma tip inside the liquid medium several micrometers away from the cell(s) to avoid mechanical damage due to tip—cell membrane surface interaction, and (ii) the targeted cell(s) were treated with the microplasma with the tip positioned outside and on top of the liquid, ~ 1 mm above the surface and about 2–4 mm from the immersed cell (Fig 1). In both cases, the cells were treated for the same plasma treatment times e.g. 10, 30, 60 and 180 s and the effects of the treatment were studied immediately and after incubation for 30 min, 3 and 24 hours afterwards. The samples were then labelled with positions based on underlying grid, so that one could always return to the initial position of the time monitored cell.

**Fig 2:**
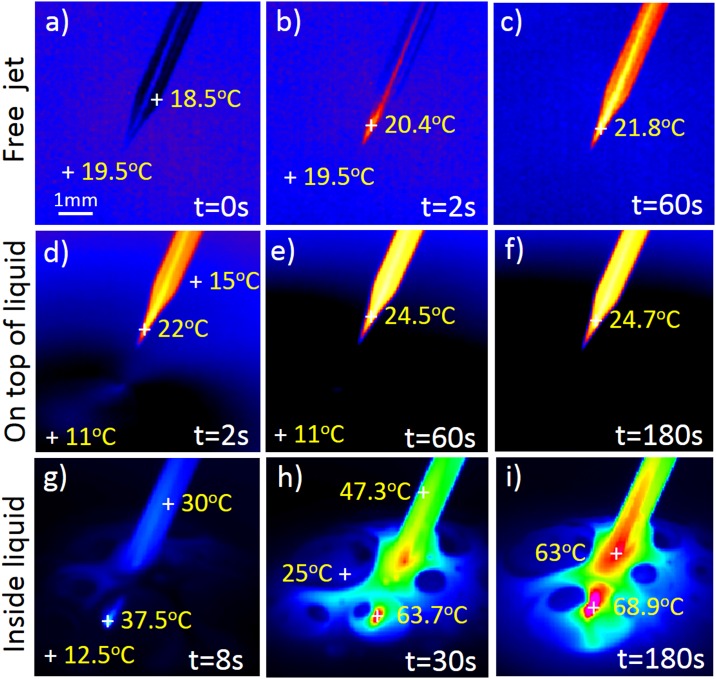
Thermal imaging of microplasma and environment in respect to treatment time. Typical images with marked temperature positions are presented for 3 cases; a)-c) free standing jet spreading into open air, d)-f) microplasma jet on the top of the liquid, and g)-i) microplasma jet inserted into liquid medium.

During plasma treatment, the environmental properties were monitored by thermal imaging using IR camera FLIR SC5000. The images recorded the tip of the glass tube with a plasma jet and zone of interaction including liquid medium. The thermal images detected were adjusted for glass emissivity (0.92), where the background temperature was adjusted to room temperature (23°C). This means that the temperature of surrounding air and the liquid shown in Fig 2 can deviate for several degrees. When doing the time evolution of the temperature in selected points, the emission coefficient was adjusted to the materials of each point; again 0.92 for the glass, and 0.96 for the pure water as approximation of the liquid medium being used. Since the liquid medium was not water but it had many additives, the emissivity was again not precise, and thus, the measurements should be taken just as an indication of the temperature change. However, the initial measured temperature of the liquid before plasma ignition was within 10–11°C of the room temperature.

### Tissue collection and processing

All tissue collection complied with the Guidelines of the Helsinki Declaration and was approved by the National Medical Ethics Committee of Slovenia, while patients signed an informed consent before surgery. The aLC-LEC explants were obtained from routine uneventful cataract surgery (20 eyes from 20 patients from Slovenia) performed by M.H. at the Eye Hospital, University Medical Centre (UMC), Ljubljana, Slovenia. All cataracts were done in elderly patients in whom the cataractogenesis was age-related and of progredient type. Lenses were dissected so that the aLC (i.e. basal lamina and associated LECs) were isolated from the fiber cells that form the bulk of the lens. After the surgery, the LEC capsules were transferred [30] to cell culture plastic glass bottom Petri dishes (Mattek Corp., Ashland, MA, USA; 3.5 cm in diameter) and cultivated *ex vivo* under adherent conditions in high glucose-containing medium (DMEM; Gibco^®^, low glucose, GlutaMAXTM supplement, pyruvate)) supplemented with 10% human serum (Sigma-Aldrich; from human male AB plasma, USA origin, sterile-filtered)and 1% Penicillin-Streptomycin (Sigma-Aldrich; Penicillin-Streptomycin with 10,000 units penicillin and 10 mg streptomycin/mL, sterile-filtered). Detailed description of the aLC tissue attachment, LEC proliferation and migration has been described previously.[7, 8] After 2–3 weeks of aLC incubation, LECs migrated from the capsule to the bottom of the Petri dish, adhered and proliferated. *Ex vivo* cultured human aLC-LECs were used throughout all of the experiments performed.

### Real-time monitoring of morphological, apoptotic and migratory changes of the cells

The cell culture medium was treated with the same APP set up as the cells before. After the exposures, Hydrogen Peroxide (H_2_O_2_) Detection Assay with the ferric-xylenol orange complex (xylenole orange, sorbitol and ammonium iron sulfate; all obtained from Sigma-Aldrich) was used with UV-Vis multiplate reader (Biotek Epoch) to determine the concentration of newly produced H_2_O_2_ in the liquid. Similarly, the nitrite concentrations were measured with standard Griess Reagent Assay (Promega; Griess reagent system). pH levels were determined as well with pH strips (Merck; pH strips).

To asses the apoptotic effect of the microplasma exposure on a treated LEC, real-time morphological observations were performed with an inverted light microscope (Axiovert S100, Carl Zeiss, AG, Oberkochen, Germany). Image acquisition was carried out by a 12-bit cooled CCD camera SensiCam (PCO Imaging AG, Kelheim, Germany). The software used for the acquisition was WinFluor (written by J. Dempster, University of Strathclyde,Glasgow, UK). Microscope objectives used were: 4x/0.10 Achroplan, 10x/ 0.30 Plan-NeoFluar and 40x/0.50 LD A-plan (Zeiss). Real-time observations of the cell morphology changes were carried out and photographed. The surrounding cells were used as controls. The criteria for selecting a region for imaging were the presence of adherent cells and intact cell morphology.

Furthermore DNA fragmentation in apoptotic cells was detected by DAPI staining of LEC samples prefixed in 4% paraformaldehyde (PFA) solution in PBS at room temperature. Immediately after this, the samples were analysed under a fluorescence microscope using standard fluorescein filter set to view 4',6-diamidino-2-phenylindole DAPI fluorescence at 460 nm and counted in three separate visual areas expressed as mean ± SD.

For immunofluorescent staining of the studied cells, fixed samples in 4% PFA were labelled by anti-Caspase 8 (goat polyclonal, Santa Cruz (sc6130), dilution 1:100) apoptosis detecting antibody, and anti-CXCR4 (rabbit polyclonal, Abcam (ab7099), dilution 1:100) cell migration detecting antibody, while nuclear staining was performed using DAPI. Fluorescent images were taken by a ZEISS Axio Observer.Z1 (ZEISS, Oberkochen, Germany) microscope. Due to scarcity of material used for immunocytochemical study and difficulty showing positive and negative controls of the stainings performed on the same cells, only inter-channel fluorescence analysis and differentiation could be performed on a same donor sample, i.e. green-labelled Caspase-8 protein was checked in the red channel for negativity, which was the case; similarly, red labelled CXCR4 protein was checked in the green channel for negativity, and that was also the case.

## Results

### Morphological changes of the cells

In order to investigate the effect of microplasma on targeted treatment of LECs, the morphological changes were studied. Before the microplasma exposure, all the cells were healthy and spindle-shaped with clear contours. Untreated LEC assumes extensively spread morphology: the cell body and cytoplasm are clearly visible. Cells adhere to the substrate in clusters, with cell extensions (filopodia) projecting toward other cells. The cytoplasm of untreated LEC appear smooth and rounded.

**Fig 3:**
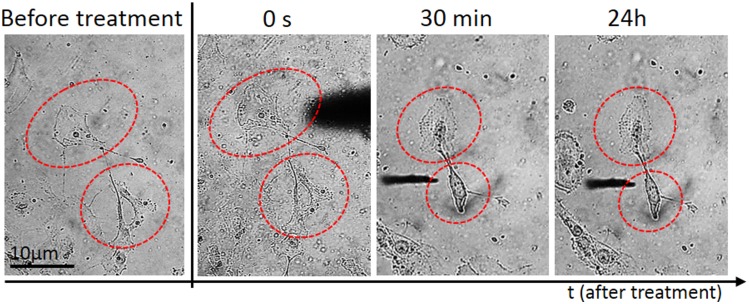
Real-time monitoring of morphological changes of aLC-LEC during indirect plasma treatment. A doublet of adherent LECs were selected and treated by the microplasma on top of the medium for 30 s. The monitored targeted cells in time after the treatment are labeled by the red dotted line.

**Fig 4:**
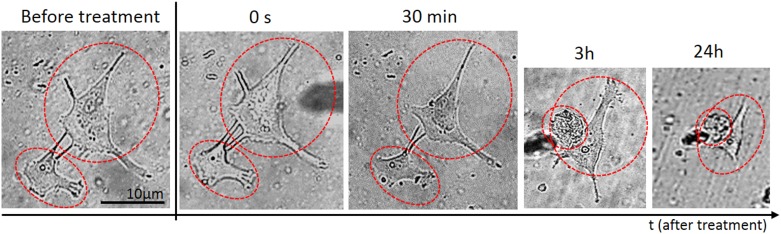
Real-time monitoring of morphological changes of aLC-LEC during direct plasma treatment. A doublet of adherent LECs were selected and treated by the microplasma directly inside the medium for 30 s. The monitored targeted cells in time after the treatment are labeled by the red dotted line.

Following plasma treatment, the LEC morphology varied greatly, but was highly dependent on the type and duration of treatment. Immediately after the treatment with the microplasma inside the medium, the affected single cell started shrinking and assumed poor morphology, indicated by a more apoptotic appearance with presence of apoptotic bodies and discernible nucleus. Weak membrane blebs appeared after 30 min, following the 30 s of plasma treatment applied on top of the medium for a twin cell system Fig 3. In comparison, the same morphological changes were observed also at plasma-treatment time of 30 s, after 30 min of incubation, when plasma was applied inside the medium (Fig 4). However, the cell membrane blebbing was also more apparent with prolonged incubation time (Fig 4; 2 h and 24 h), whereas in case the first case of Fig 3 almost no changes are observed in prolonged incubation. On the other hand, the non-treated control cells were unaffected and healthy during the entire incubation periods. These distinctive morphological changes were the simplest indicators of increase in cell death.

Typically, Fig 4 where a double-LEC system was present and following plasma treatment for 30 s inside the medium, the large difference in cell morphology appeared after 30 minutes, when compared to control untreated LECs: the cells were both shrunk and more rounded, with predominantly raised nuclear region. The stress bundles of actin filaments could also be seen, particularly around the cell edges and some rounded features corresponding to organelles as seen under the surface by light microscopy. Compared to the untreated LECs, damaged parts of the cell membrane and other cellular components could be observed. The cytoplasm of the cell seemed very rough and dense, which is another consequence of plasma treatment. More pronounced changes like cellular shrinking and alterations of the cytoplasmic structure could be observed on the LEC 3 h after plasma treatment. The targeted-cell treatment with microplasma exhibited multiple perforations on the membrane (which could not be noticed on the untreated cells) and loss of nuclear structures (e.g. nucleoli) (Fig 3). After longer incubation time (24 h following plasma treatment), the observed effects were similar to those after 3 h, although the changes on cell shape, cytoplasmic and nuclear, were even more pronounced Fig 4.

**Fig 5:**
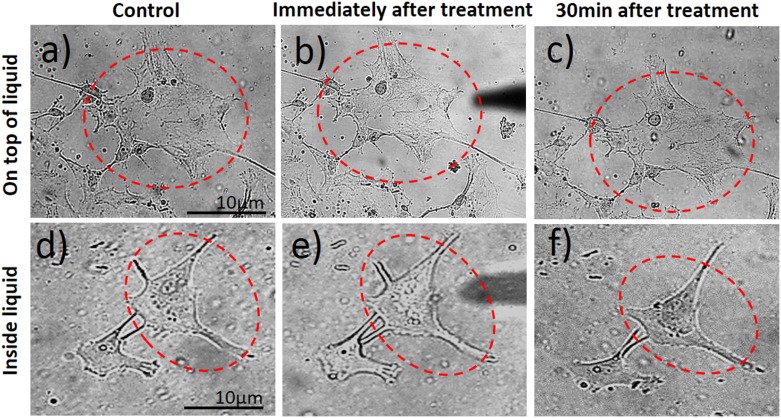
Comparison of the morphological changes evoked in a LEC. Indirectly and directly treated with plasma are being shown for the same plasma treatment time of 30 s.

**Fig 6:**
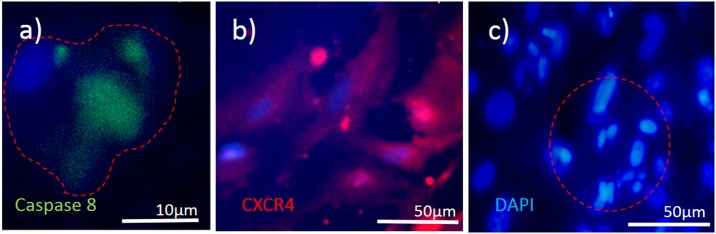
Cell death analysis and migration potential of the treated cells. 3 representative cases are being shown: a) immunostaining againt expression of Caspase 8 (an apoptosis marker), and b) CXCR4 (cell migration marker); c) DAPI stained cells (marker for DNA fragmentation) for assessment of cell death. The red-dotted line presents boundary of a single cell in a). while in c), it presents the treated area of impact with apoptotic bodies being formed.

If we compare the same treatment of randomly selected LEC using microplasma with the same microtip, but on top of the medium, delayed and sometimes varied results could be observed (Fig 5). Fig 5b and 5c show that the treated cell were not affected immediately after 30 s exposure to microplasma. The cells maintained healthy morphology after 30 min. The same treatment time of a doublet of cells inside the medium resulted in serious alteration of their morphology (Fig 5e and 5f). However, there are important differences of longer incubation time when compared to control untreated LEC (as observed with the shorter incubation treatment). Differences on the cell membrane could be observed after 30 s indirect plasma treatment of LECs. It is noteworthy that the membrane blebbing was observed 3 h after the plasma exposure (S1 Fig), which is another morphological change reminiscent of apoptosis, thus confirming the occurrence of cell death in the targeted treatment of cells after longer incubation time. Induction of apoptosis could be shown in the targeted LEC by the expression of Caspase 8 (marker of apoptosis) in a population of LECs which showed migrating (CXCR positive) properties (Fig 6). Since the nuclear changes occur relatively late in the process of apoptosis, no obvious difference could be seen even at 3 h after the microplasma treatment. However, 24 h after the plasma exposure, the nucleus of the treated cell became brighter or lense dense compared to the non-treated cell, while nuclear condensation could be observed in the treated cell (S1e Fig).

**Fig 7:**
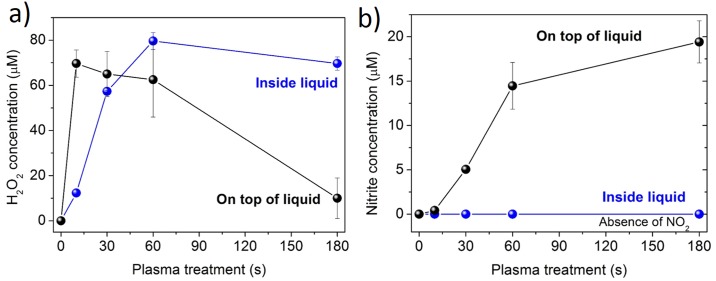
The initiated liquid chemistry with microplasma treatment on top or inside the liquid medium. a) Hydrogen peroxide and b) nitrite concentrations are being shown in respect to different treatment times.

Cell morphological changes leading to apoptosis, especially after longer incubation time are probably results of changes done by ROS which create H_2_O_2_ as well as nitrites and nitrates (Fig 7), which will be discussed later. These changes become more pronounced in longer exposure times of the liquid.

### Influence of plasma on the liquid medium

The gaseous plasma which interacts with medium modifies its properties due its interaction with liquid surface interface (on top of the liquid), as well as surrounding environment by creation of vapors (inside the liquid). The products of these reactions are in our case peroxides and nitrites. The concentrations of H_2_O_2_ progressively increased after the plasma treatments of the liquid medium (Fig 7a). Concentrations obtained after 30 s, 60 s and 180 s of treatment were significantly higher compared to control. However, much different trend was observed when the media was treated on the surface: concentrations first showed increasing tendency, which was later followed by a drop in values. The highest H_2_O_2_ concentration was observed at 60 s plasma treatment performed in the liquid medium.

On the other hand, when the cell culture medium was treated in the liquid, no nitrites were detected after the exposure, since there was no interaction of plasma with surrounding air (Fig 7b). In contrast, the nitrate concentrations gradually increased with longer exposure times. Concentrations obtained after 60 s and 180 s were significantly higher compared to control. Interestingly, the pH level remained almost constant under all treatment modalities (around value 8).

### Plasma versus mechanical effect of the gas flow

**Fig 8:**
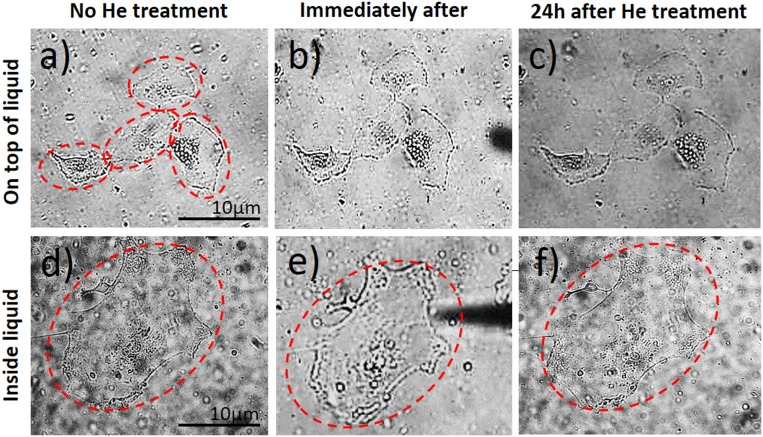
Effect of He gas flow on the cell morphological responses. Indirectly and directly treated with plasma are being shown.

In order to make sure that the treatment is the result of ROS generated by the microplasma discharge in comparison to the He gas flow-related effects, a set of control experiments were performed only with He gas. Specifically, to elucidate the effects of the plasma exposure versus the mechanical related effects, the LECs were treated using the same tips as shown in Fig 1 for only mechanical stimulation of gas. Importantly, the single cell treated with He gas exhibited no morphological changes and underwent no apoptosis, when directly or indirectly treated with plasma (Fig 8). Therefore, it can be concluded that the observed apoptotic response in the cells is indeed likely related to the plasma-generated species rather than a mere mechanical effect of moving the fluid on attached cells and due to the gas flow.

### Effect of the plasma dose on targeted treatment of LECs

**Fig 9:**
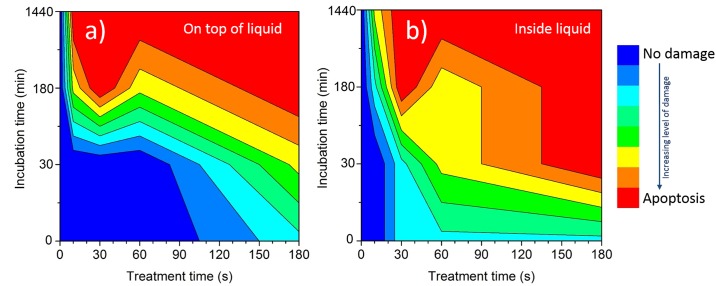
The morphological changes connected to the apoptosis induction in the cells in respect to microplasma treatment over a given time and consequent incubation of cells for a) indirect and b) direct treatments. The legend shows multiple potential apoptosis stages, which are highly likely for certain treatment after 24h, which are medium value of optical and fluorescent observation taking under consideration Gaussian distribution of results.

The results of plasma dose which is directly related to treatment time in respect to direct or indirect cell treatment are summarized in Fig 9. During direct treatments the cell effects were quickly pronounced and lead to cell damage. However, in the case of indirect treatments the effects on cells are delayed and longer treatments are needed. The longer the plasma exposure, the stronger was the effect on the targeted-cell morphology, immediately after the treatment or later after incubation for 30 min or more. This trend was observed in both cases, when treatment was performed on top of liquid or inside the liquid medium. Moreover, when the targeted-cells were treated inside the medium, the effect of plasma on the treated cells was significantly stronger than when treated on top of the medium.

The results of systematic treatments of cells with the liquid interface, when on top of the liquid medium are shown in S1 and S2 Figs. As can be seen, microplasma induced the LEC to undergo apoptosis in a dose-dependent manner. The effect of plasma on LECs is only observed for incubation times above 3h under current experimental conditions (treatment times of 30 and 60 s).

When treatment was performed inside the medium (S3–S5 Figs) shorter treatment times were needed to observe morphological changes as compared to treatments on top of the medium. Interestingly, already at the short treatment times, the microplasma induced morphological changes in LEC immediately after the treatment.

### Effects of plasma on the surrounding LECs

To clarify the effect of the microplasma exposure, the targeted-cells undergoing plasma treatment were compared to the surrounding cells. A well-defined boundary between plasma-treated cells and untreated cells could be observed. The most obvious case for this is seen in Fig 6c: the stained cells with DAPI and a marked region of apoptotic bodies after 30 s treatment inside the liquid can be seen, while the marked region is surrounded by untreated cells. Importantly, just the plasma-treated cells were induced to undergo cell death, while the neighbouring cells were not affected significantly. The effect of plasma on surrounding cells was slightly stronger when treatment was performed on top of the medium: this can clearly be seen from the 3 h long examinations. Although sometimes the surrounding cells were not strongly affected, some cells in the close vicinity still changed their morphology. However, the major problem is always mixing of the liquid medium during the treatments or after. The mixing is driven by the blowing gas, which enables migration of newly created products from the plasma-liquid interaction. These products might significantly affect the cells after longer incubation times, but their role cannot be confined.

### Other physical effects on cells

One of the most significant effects that may influence LECs is localized temperature generated by microplasma. For this reason, a sequence of experiments was performed, where the system was monitored by IR camera, when the microplasma was applied as a free jet plume in open air without any interaction with a surface, as well as the top or inside the liquid. The time series of images were taken for all measurements and most specific cases are presented in Fig 2a–2i. The results for free operating microplasma jet and microplasma on top of the liquid medium are similar: in both cases, following plasma ignition, the temperature of the tip increased to maximum 5°C for 180 s operation. This is mostly a result of pure He plasma species interacting with the surrounding walls, which are simultaneously cooled by gas flow. The liquid medium temperature is almost constant during indirect microplasma treatment (Fig 2d–2f). On the contrary, the liquid was significantly heated during direct treatment inside the liquid (Fig 2g–2i). Here, the localized area was heated to almost 70°C after prolonged operations, which probably occurred due to the created vapours when plasma is in contact with liquid. In cellular terms, this means that an already significant influence of temperature upon the cells could be generated, which can lead to their apoptosis.

An important factor that can sometimes influence the cell viability and physiological state is also UV radiation many times released from the plasma source. This should be taken under consideration. The generated microplasma in pure He has typical major emission lines in the range between 388 nm to 667 nm. However, in our case, we deliberately neglected this, especially in light of the temperature effect measured for inside liquid treatments, whereas on the top of the liquid, there is strong absorbing layer of organic fluid with proteins which we believe can reasonable well protect the cells based on UV-VIS measurements spectra of liquid. Another potential effect on the cells vitality can be also created by the electric field around the electrode tip. If electrode wire tip is considered to be a cylinder, then the calculated electric field at top of the electrode is ~8·10^2^ V/cm. However, to observe any apoptotic effects upon the cells from electroporation science point of view, a pulsed electric field above 10^3^ V/cm would be needed. Taking into consideration that the nearest cells without liquid interface are at a distance of 2 to 3 mm from the tip, where the electric field falls with the distance, the field can then be taken as negligible to influence the cell vitality.

## Discussion

The present study illustrates the interaction of μAPPJ targeted to the cell surface of *ex vivo* cultured aLC-LECs when treated by microplasma inside and on top of the culture medium. The effect of plasma on the cell morphology and cell death is therefore evaluated, as well as its effect on the surrounding cells and the mechanical effects of the gas flow. It is shown that plasma affects only a limited number of treated LECs, which morphology over time changes to apoptotic, while leaving the surrounding LECs unaffected. Furthermore, no mechanical effect of the gas flow on the cell morphology could be observed, which proved that the observed viability changes in the LECs were indeed generated by the ROS species induced by the plasma.

Based on the changes seen in the targeted-cell morphology following plasma treatment, it can be concluded that charged and reactive species produced in the plasma and coming in direct contact with the treatment target, play a key role in the biological mechanisms, probably initiating and catalysing production of hydroxides, nitrites and nitrates, which might cause induction of apoptosis in later stages. The primary target of the direct plasma treatment is the cell membrane. Past the membrane, the plasma-related or physical mechanisms cease and the biochemical interactions seem to take place such as DNA damage and nuclear condensation.[10]

No change in the pH level is usually observed when plasma treated liquid medium contains a buffer [31], which was the case in our experiments, where the major part of the DMEM culture medium is composed of a phosphate buffered saline (PBS). The contact of plasma with the surrounding air plays a major role in the formation of nitrites, since no concentrations of them could be detected when the treatments were performed inside the liquid. Under such conditions, only H_2_O_2_ was formed, which is probably a result of the plasma interaction with the water molecules. On the other hand, the interaction between plasma and air results (on top of the liquid) in the formation of nitrogen and oxygen species which are later diluted in the liquid also as peroxides and nitrites. [31]

A careful investigation of the modified liquid and targeted cell changes revealed that most processes occur on the cell membrane, e.g. the phospholipid bilayer of the cell. The key processes occurring on the membrane are probably peroxidation of lipids and polysaccharides due to oxidative species and charges (OH^-^, OOH, O_2_^-^, e^-^). However, there are also effects of nitrites and nitrates, which are created in smaller quantities and only during indirect treatments. The effect is chemical and highly dependent on the amount of medium and on the chemical composition of the medium surrounding the cells. The bigger the volume of the medium, a weaker effect on the treated biological species is observed.[10] In our experiments, the volume of the treated medium was constant (~ 2.0 cm^3^) for all performed treatments. The influence of medium and their modifications which surrounds the cells plays an important role, especially in later stages, where the much stronger effect of direct plasma treatment is observed on the targeted-cells due to the interaction of plasma helium species (He* and He^+^) with cells and created vapours from the surrounding liquid (O_2_^-^, H^-^, OH^-^, H_2_O^-^) and in comparison to indirect treatment. The treated medium interface may minimize the effect of the plasma due to dispersal of charged particles especially (e^-^)_aq_, (OH^-^)_aq_ or NO_2_^-^, NO_3_^-^ as well as some reactive species inside the medium [32]. In this case, the role of plasma species created in bulk plasma or created vapours is reduced, and as result different chemical reactions take place in the liquid phase compared to gas phase. Additional difference between both conditions is also that, in indirect treatments, He bulk plasma interacts with ambient air and creates more nitrogen or nitrogen containing species, which might play important role in the structure or the created vapour or in the penetration into liquid media (Fig 1). Dispersal by the charged particles such as electrons and ions that are generated by the gas discharge, and initially formed near the electrode region, move to the nozzle of the tip and are finally discharged into the ambient air. While the generated plasma and neutral He gas passes through the narrow nozzle of the tip, the linear velocities of their flow abruptly increases with a decrease of the inner diameter of the tip. For this reason, the charged species are better spread into the liquid (by Henry’s law) due to diffusion and turbulences occurring at the surface of the medium and due to their velocity difference at the boundary between the tip nozzle and the ambient air [33, 34]. This can make a difference in plasma treatments either influencing larger zone of plasma impact or in indirectly diminishing effects of plasma to targeted cells by dispersion of species through the entire liquid. On the other hand, the discharge created inside the liquid creates vapours which enable better energy-heat transfer and can significantly heat the several mm^2^ zone and potentially damage larger number of cells. The temperatures are significant and increased to several tens of degrees Celsius. All these effects have to be considered in the evaluation of plasma results and prolonged treatments, where H_2_O_2_, nitrites and nitrates are being generated.

In order to assess the differences in morphology and the effect of plasma treatment time, the morphological appearances of the adherent post-operatively cultured LECs were studied right after the treatment, or 30 min, 3 h and 24 h after plasma treatment. Microscopic images revealed important information on the shape and cell adhesion before and after plasma treatment with substantial differences being observed between the samples. Specific impact of the plasma on the treated cell shape and morphology after longer 3 h and 24 h of incubation could be shown, with cell morphology being significantly changed only after longer treatments, which also suggests limited effects of temperature during direct treatments. On the other hand, the results suggest that surrounding LECs remain unaffected. Furthermore, important differences between direct and indirect treatments could be observed—the plasma exposure time needed to reach observable morphological changes on targeted LECs was 6-times shorter under direct plasma treatment. These results show that differences between direct and indirect treatments are not only important for fast induction of apoptosis, but also suggest the important role the medium has on the viability of plasma treated LECs.

Under direct treatment of targeted LECs inside the medium, the plasma comes in direct contact with the cells and achieves the desired effect in orders of magnitude faster than indirect application, where plasma is separated from the treated target by the medium (plasma treatment of LECs performed on top of the medium). Likely, this effect can be primarily attributed to the bulk He plasma and created vapours along with time dependent heating. Thus, the key role in plasma–cell interaction is played probably by the ions or ionized molecules being generated and specifically, the positive and negative ions which have a relatively similar effect. The charged species being generated pose a chemical effect which is not related to the physical phenomena such as stress (mechanical stress induced by the moving fluid), ion bombardment damage, or thermal effects. Ions catalyze oxidation processes both inside and outside of the cell, which explains why they are able to have greater effect than neutral active species.[18]

In the work by Dobrynin *et al*. [10], the role of water in direct and indirect plasma inactivation of bacteria was studied. The first method was ‘direct’ application of plasma to bacteria, where the bacteria were used as a second active electrode—plasma was bound between the dielectric surface of the powered electrode and the surface of the bacteria being treated; the second method was ‘indirect’ application of plasma, where plasma was separated from the bacteria by a grounded metal mesh and gas was blown through the discharge to carry active species outside of the plasma. It was shown that direct application of plasma yields roughly a two orders of magnitude improvement in the rate of bacterial inactivation as compared to indirect application, even when the plasma is removed from the tissue by a fraction of a millimetre.[35] Similarly, we observed that plasma treatment inside the medium, achieved much stronger effect of the plasma on the treated LECs. Although prokaryotic and eukaryotic cells are very different regarding the cell structure and morphology, the effect of plasma treatment on both may be very similar.

A likely mechanism of plasma interaction with cell is through the membrane, as it is the primary cell barrier on its way of penetration. Different mechanism is also possible such as formation of small pores due to high local electric fields proposed by Joshi *et al*. [36, 37], which may lead to either cell leakage or a pathway for entrance of radicals into the cell. These pores, depending on their size, may re-close rapidly and the cell would appear intact under the microscope while the damage done may be permanent and lead to subsequent cell death. Another potential mechanism is through the use of the cell’s own enzymes: ions present in the solution, especially ions introduced from plasma, may activate the secondary messenger system that amplifies any external signal [38]. This way, plasma may be able to alter the behaviour of the cell by simply altering the ionic strength of the solution in the treated media [12, 39].

Interaction of plasma with DNA also should be taken into account. Arjunan *et al*. summarized the effects of APP on both isolated and cellular DNA [40], whereas O’Connell *et al*. reported on cold APPJ interactions with plasmid DNA. In the latter, the atomic oxygen density was correlated to the rates of single and double strand DNA breaks' formation [41]. Moreover, Lazović *et al*. compared the effects of plasma to gamma irradiation in terms of DNA damage [42]. Generally, peroxidation of phospholipid bilayer is also known to cause cellular death through a chain process leading to the formation of DNA adducts [43–45]. Interestingly, these defects in DNA are relatively easily repaired by mammalian cells [46]. Other important mechanism of plasma action on the cell is through formation of ROS directly in the vicinity of the DNA molecules inside the cell nucleus [47, 48]. The ROS of interest are hydroxide (OH^-^), H_2_O_2_ and a superoxide anion (O_2_^−^). ROS are not generated at the vicinity of a DNA molecule, but are rather transported to it through a series of mechanisms already present in the cells. Hydroxyl radicals can react with nearby organic molecules, leading to chain oxidation and thus destruction of DNA as well as cellular membranes and other cell components [10]. Therefore, charges generated during plasma treatment may have an effect on bacteria and cells in solution due to the oxidation and peroxidation chain reactions they can catalyze; the same may be a reason for the difference in treatment times required to inactivate bacteria versus those needed to achieve cellular damage.

The true mechanism of atmospheric plasma on cells, especially when treated inside the medium, remains to be elucidated. However, the present study provides further data on the effect of cold plasma on a LEC viability by studying the cell morphology before and after plasma treatment inside and on top of the medium. Because of the extreme complexity in plasma-cell interaction, it is difficult to determine which mechanisms the cell uses to protect itself against stress (e.g. plasma treatment) and which signalling pathways are initiated inside the cell, although some implications for what is happening inside LECs after direct and indirect plasma treatment can be assumed. While the response of a single-cell treated inside the medium is significantly faster and more pronounced than that of a single-cell treated on top of the medium, we can speculate different initial signalling pathways get activated after plasma treatment under each condition.

The present data will serve to better define the complex interaction between cold plasma and the cell membrane surface and aid in the design of future experiments. To our knowledge, this is the first report of targeted cell treatment with microplasma inside a medium, which is more effective in inducing cell death when applied directly. A significantly shorter time is needed for a single-cell to undergo apoptosis and thus effective microplama single-cell therapy under such conditions. Moreover, plasma does not influence the large number of surrounding cells, which is of course beneficial and favourable for achieving highly localized-cell therapy.

Our results show that with a precision of a single cell level, plasma needle and plasma treatment can be used to selectively kill LECs that remain in the capsular bag after cataract surgery that can otherwise re-colonize the posterior lens capsule and create PCO, thus, obstruct the visual axis and contribute to light scattering and decreased visual acuity. Even more, our results suggest that μAPPJ application can achieve medically relevant therapeutic effects, which can potentially be applied in wide range of eye and other medical conditions.

## Conclusions

Despite the small inner diameter and very low gas flow rate, a microplasma releasing device can induce apoptosis in LECs in a dose-dependent manner under both, direct and indirect treatment conditions. The microplasma can be confined to the small (~3 μm) volume around the tip of the needle, which can be positioned in any specific area by using a micromanipulator. The power delivered to the cell is very small (1 W) yet sufficient to induce apoptosis, without affecting a large number of neighbouring cells, even within small clusters of closely contacting cells. A boundary region between plasma-treated and untreated cells could be observed on the order of cellular dimensions. We hereby demonstrate that inside the medium, the effect of microplasma on a treated targeted-cells is 6-times stronger than on top of the medium. Immediate morphological changes could be observed already after 30 s of direct treatment with microplasma, in comparison to indirect treatment, when immediate changes in morphology of the treated single-cell were observed only after longer treatment periods. These findings support the notion that direct treatment with microplasma inside the medium is more effective, as the treatment time needed for immediate response of a cell is significantly shortened. This variation allows the direct application of a microplasma jet device for precise use for single cell manipulation to selectively induce demise in LECs.

Although these results are potentially promising, many unanswered questions and gaps in understanding of cell-plasma interaction remain. The interaction mechanisms of microplasma inside and on top of the medium with the cells, and the potential reasons for the observed significant differences presented here, are an open question requiring deeper understanding before microplasma applications *in vivo* or for operative-therapeutic purposes. A logical step to follow in these targeted-cell plasma experiments may be to investigate the communication pathways and critical distances between the cells in different cluster configurations as a ground for testing collective tissue and cluster cell responses to plasma treatments.

## Supporting Information

S1 Fig Real-time monitoring of morphological changes at the cell level in LEC. A targeted adherent LECs were selected and treated by the microplasma *on top of the medium* for **30 s**. The monitored cell in time after treatment is labeled by the red dotted line.
(TIFF)

S2 Fig Real-time monitoring of morphological changes at the cell level in LEC. A targeted adherent LECs were selected and treated by the microplasma *on top of the medium* for **60 s**. The monitored cell in time after treatment is labeled by the red dotted line.
(TIFF)

S3 Fig Real-time monitoring of morphological changes at the cell level in LEC. A targeted adherent LECs were selected and treated by the microplasma *inside the medium* for **10 s**. The monitored cell in time after treatment is labeled by the red dotted line.
(TIFF)

S4 Fig Real-time monitoring of morphological changes at the cell level in LEC. A targeted adherent LECs were selected and treated by the microplasma *inside the medium* for **60 s**. The monitored cell in time after treatment is labeled by the red dotted line.
(TIFF)

S5 Fig Real-time monitoring of morphological changes at the cell level in LEC. A targeted adherent LECs were selected and treated by the microplasma *inside the medium* for **180 s**. The monitored cell in time after treatment is labeled by the red dotted line.
(TIFF)
