# Supplementary figures and images for: Microplasma Induced Cell Morphological Changes and Apoptosis of Ex Vivo Cultured Human Anterior Lens Epithelial Cells – Relevance to Capsular Opacification
Source: PLoS One. 2016 Nov 10;11(11):e0165883. doi: 10.1371/journal.pone.0165883 (PMC5104483; doi:10.1371/journal.pone.0165883)

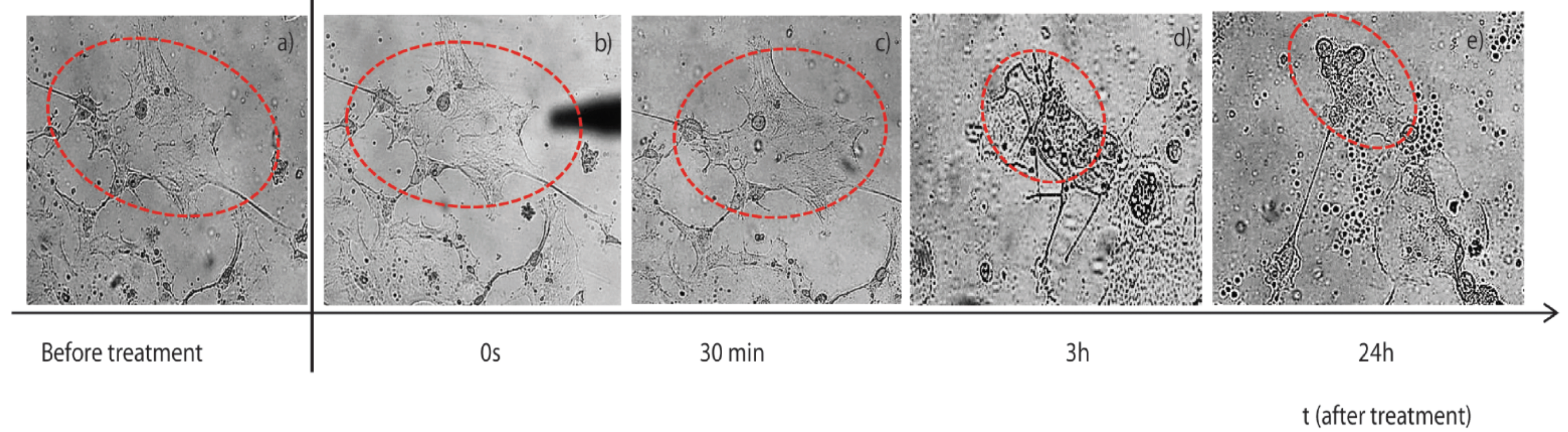

Supplement: S1 Fig — A targeted adherent LECs were selected and treated by the microplasma on top of the medium for 30 s. The monitored cell in time after treatment is labeled by the red dotted line. (TIFF) [file pone.0165883.s001.tiff]

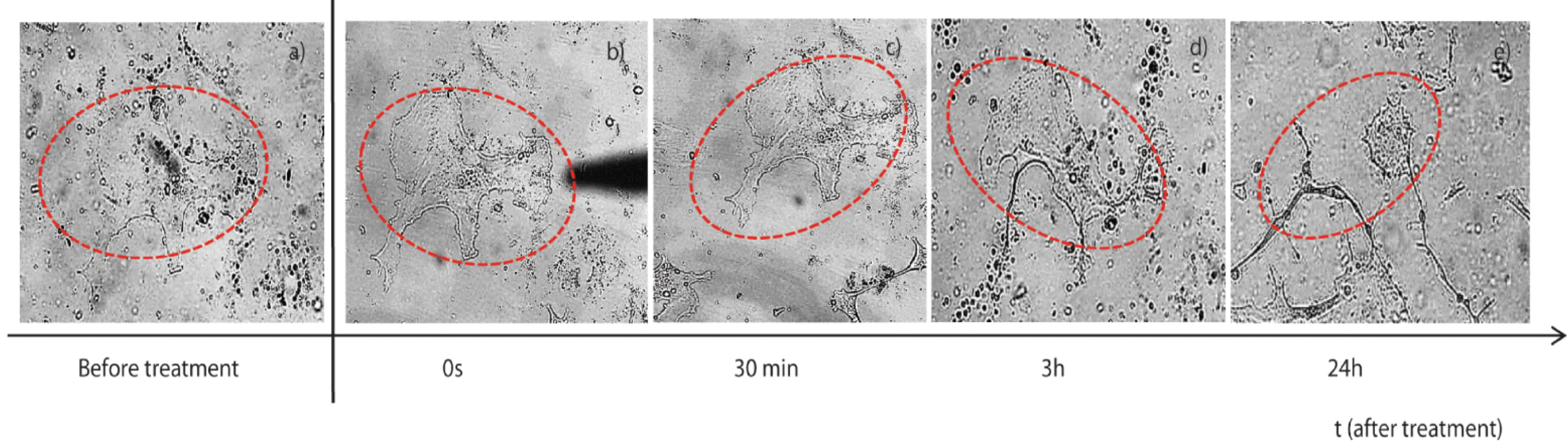

Supplement: S2 Fig — A targeted adherent LECs were selected and treated by the microplasma on top of the medium for 60 s. The monitored cell in time after treatment is labeled by the red dotted line. (TIFF) [file pone.0165883.s002.tiff]

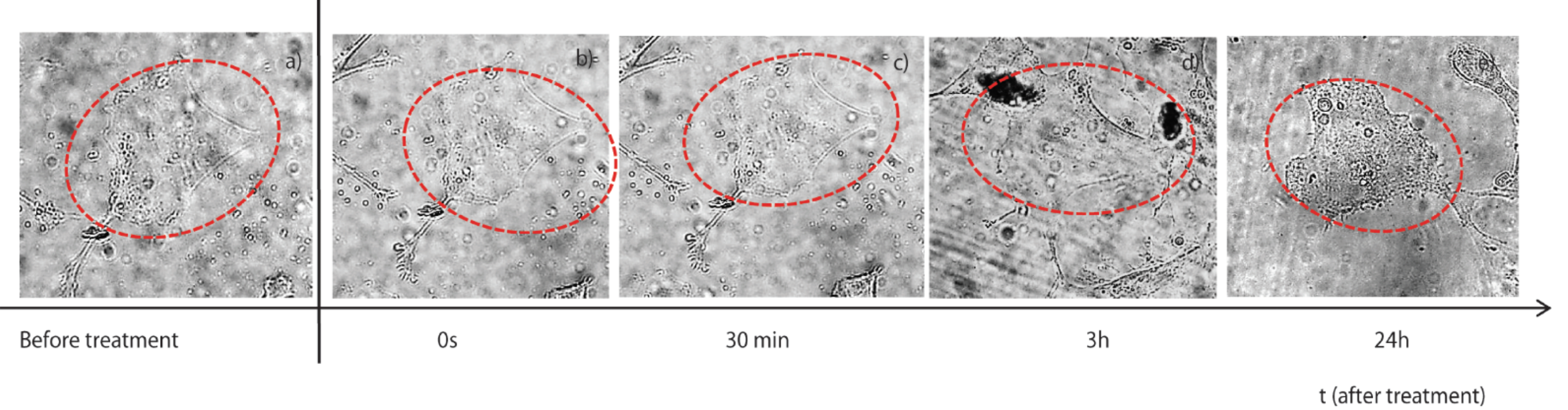

Supplement: S3 Fig — A targeted adherent LECs were selected and treated by the microplasma inside the medium for 10 s. The monitored cell in time after treatment is labeled by the red dotted line. (TIFF) [file pone.0165883.s003.tiff]

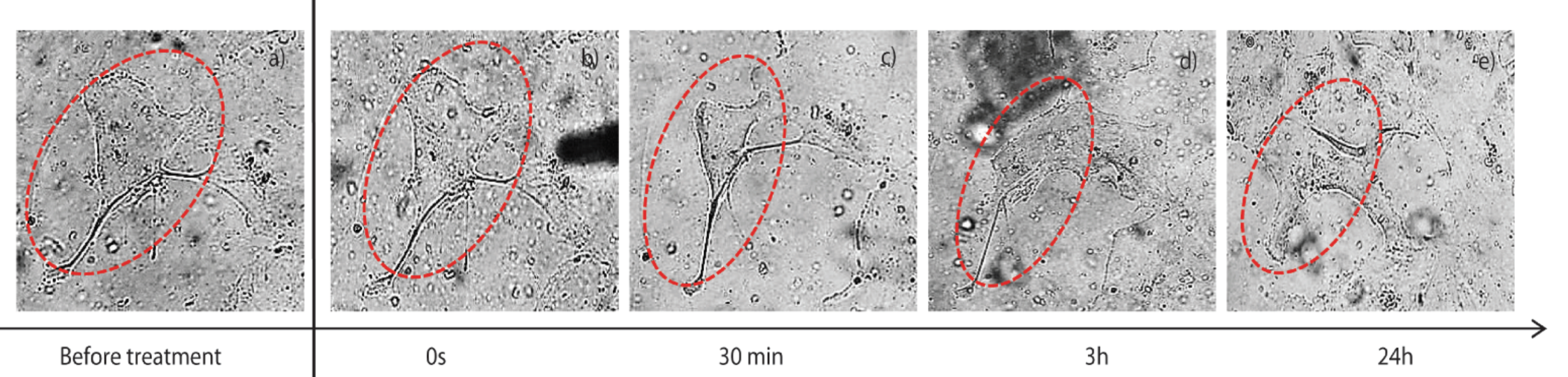

Supplement: S4 Fig — A targeted adherent LECs were selected and treated by the microplasma inside the medium for 60 s. The monitored cell in time after treatment is labeled by the red dotted line. (TIFF) [file pone.0165883.s004.tiff]

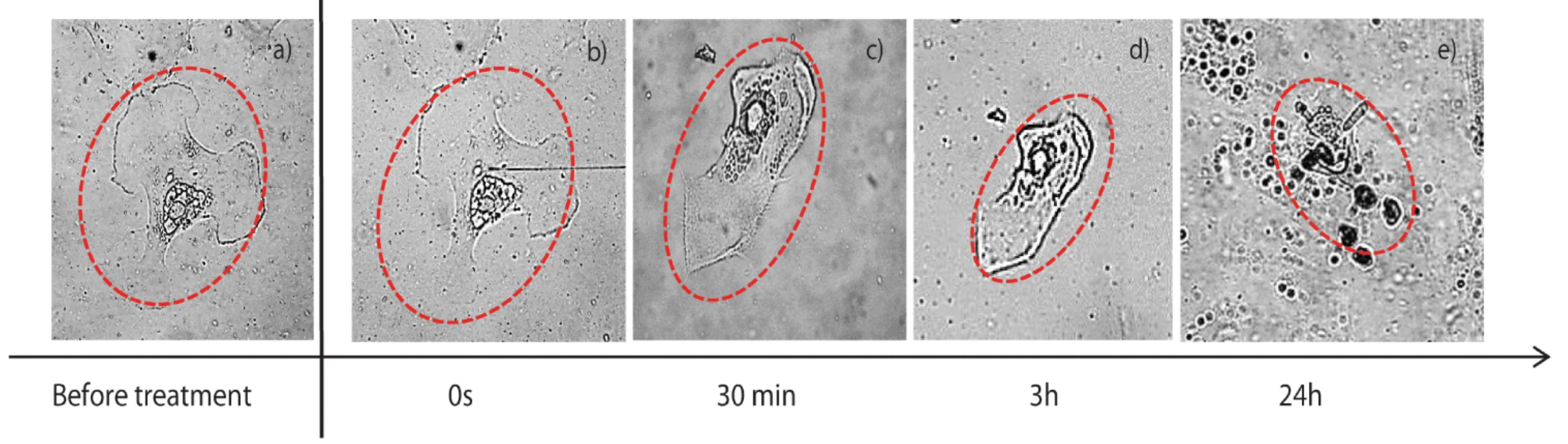

Supplement: S5 Fig — A targeted adherent LECs were selected and treated by the microplasma inside the medium for 180 s. The monitored cell in time after treatment is labeled by the red dotted line. (TIFF) [file pone.0165883.s005.tiff]
